# Occupational Exposure to Benzene and Non-Hodgkin Lymphoma in a Population-Based Cohort: The Shanghai Women’s Health Study

**DOI:** 10.1289/ehp.1408307

**Published:** 2015-03-06

**Authors:** Bryan A. Bassig, Melissa C. Friesen, Roel Vermeulen, Xiao-Ou Shu, Mark P. Purdue, Patricia A. Stewart, Yong-Bing Xiang, Wong-Ho Chow, Tongzhang Zheng, Bu-Tian Ji, Gong Yang, Martha S. Linet, Wei Hu, Heping Zhang, Wei Zheng, Yu-Tang Gao, Nathaniel Rothman, Qing Lan

**Affiliations:** 1 Occupational and Environmental Epidemiology Branch, Division of Cancer Epidemiology and Genetics, National Cancer Institute (NCI), National Institutes of Health (NIH), Department of Health and Human Services (DHHS), Bethesda, Maryland, USA; 2 Institute for Risk Assessment Sciences, Utrecht University, Utrecht, the Netherlands; 3 Division of Epidemiology, Department of Medicine, Vanderbilt Epidemiology Center, Vanderbilt University School of Medicine, Nashville, Tennessee, USA; 4 Occupational and Environmental Epidemiology Branch, Division of Cancer Epidemiology and Genetics, NCI, NIH, DHHS, Bethesda, Maryland, USA; 5 Stewart Exposure Assessments LLC, Arlington, Virginia, USA; 6 Shanghai Cancer Institute, Renji Hospital, Shanghai Jiaotong University School of Medicine, Shanghai, China; 7 Department of Epidemiology, The University of Texas MD Anderson Cancer Center, Houston, Texas, USA; 8 Department of Environmental Health Sciences, Yale School of Public Health, New Haven, Connecticut, USA; 9 Radiation Epidemiology Branch, Division of Cancer Epidemiology and Genetics, NCI, NIH, DHHS, Bethesda, Maryland, USA; 10 Department of Biostatistics, Yale School of Public Health, New Haven, Connecticut, USA; 11 Department of Epidemiology, Shanghai Cancer Institute, Shanghai Jiaotong University, Shanghai, China

## Abstract

**Background:**

The association between benzene exposure and non-Hodgkin lymphoma (NHL) has been the subject of debate as a result of inconsistent epidemiologic evidence. An International Agency for Research on Cancer (IARC) working group evaluated benzene in 2009 and noted evidence for a positive association between benzene exposure and NHL risk.

**Objective:**

We evaluated the association between occupational benzene exposure and NHL among 73,087 women enrolled in the prospective population-based Shanghai Women’s Health Study.

**Methods:**

Benzene exposure estimates were derived using a previously developed exposure assessment framework that combined ordinal job-exposure matrix intensity ratings with quantitative benzene exposure measurements from an inspection database of Shanghai factories collected between 1954 and 2000. Associations between benzene exposure metrics and NHL (*n* = 102 cases) were assessed using Cox proportional hazard models, with study follow-up occurring from December 1996 through December 2009.

**Results:**

Women ever exposed to benzene had a significantly higher risk of NHL [hazard ratio (HR) = 1.87, 95% CI: 1.19, 2.96]. Compared with unexposed women, significant trends in NHL risk were observed for increasing years of benzene exposure (*p*_trend_ = 0.006) and increasing cumulative exposure levels (*p*_trend_ = 0.005), with the highest duration and cumulative exposure tertiles having a significantly higher association with NHL (HR = 2.07, 95% CI: 1.07, 4.01 and HR = 2.16, 95% CI: 1.17, 3.98, respectively).

**Conclusions:**

Our findings, using a population-based prospective cohort of women with diverse occupational histories, provide additional evidence that occupational exposure to benzene is associated with NHL risk.

**Citation:**

Bassig BA, Friesen MC, Vermeulen R, Shu XO, Purdue MP, Stewart PA, Xiang YB, Chow WH, Zheng T, Ji BT, Yang G, Linet MS, Hu W, Zhang H, Zheng W, Gao YT, Rothman N, Lan Q. 2015. Occupational exposure to benzene and non-Hodgkin lymphoma in a population-based cohort: the Shanghai Women’s Health Study. Environ Health Perspect 123:971–977; http://dx.doi.org/10.1289/ehp.1408307

## Introduction

Benzene is a common occupational solvent that has been used in multiple industries, particularly as a chemical intermediate in the production of plastics and in rubber manufacturing, and has been used as a solvent in common consumer products worldwide (Wilbur et al. 2008). Moreover, exposure to benzene is ubiquitous in the general population because it is present in gasoline, automobile emissions, and cigarette smoke (Wilbur et al. 2008). There has been continued concern regarding potential adverse health effects arising from airborne benzene exposures, particularly in light of evidence that benzene is hematotoxic at exposure levels as low as < 1 ppm (Lan et al. 2004; Qu et al. 2002) and is associated with an increased risk of myelodysplastic syndrome at relatively low exposure levels (Schnatter et al. 2012). In China, benzene is regularly used in manufacturing industries, and historically high levels of occupational benzene exposure have been reported, with a number of reports describing cases of benzene poisoning in exposed Chinese workers (Liu et al. 2009).

Exposure to benzene, which is classified by the International Agency for Research on Cancer (IARC) as a Group 1 carcinogen, has been concluded to cause acute myeloid leukemia (AML), based in part on results from several case–control and occupational cohort studies in a variety of human populations (IARC 2012). In addition, a variety of other adverse health effects associated with benzene have been observed in animal and human studies, including developmental, neurological, and hematopoietic toxicities (Wilbur et al. 2008). Benzene is also a suspect lymphomagen based on positive associations with non-Hodgkin lymphoma (NHL) overall or with specific NHL subtypes observed in some occupational cohort and case–control studies, although the strength of the evidence is not as strong as the association with AML (Baan et al. 2009; IARC 2012). In particular, a significant exposure–response association with NHL was previously identified for benzene exposure in a large occupational cohort study of benzene-exposed workers in China (Hayes et al. 1997). Several more recent case–control studies conducted in the United States and in Europe have provided some additional evidence for an association with NHL overall (Dryver et al. 2004; Fabbro-Peray et al. 2001) or with specific subtypes including follicular and diffuse large B-cell lymphoma and chronic lymphocytic leukemia (CLL) (Costantini et al. 2008; Miligi et al. 2006; Wang et al. 2009). Based in part on these epidemiologic studies, an IARC working group recently concluded that there is evidence for a positive association between benzene exposure and NHL risk in humans (IARC 2012).

To our knowledge, no previous studies have evaluated the association between benzene exposure and NHL in the context of a population-based prospective cohort study with diverse occupational histories. Recently, a unique framework for assessing historical benzene exposure was developed and applied in a population-based cohort study of Chinese women living in urban Shanghai (Friesen et al. 2012). This approach combined a job-exposure matrix (JEM) with short-term area air measurements of benzene in factories in Shanghai over the period 1954–2000, and has been demonstrated to increase contrast in exposure levels between different industries and jobs, as well as over time, compared with using the JEM alone (Friesen et al. 2012). Here, we applied this exposure assessment framework to evaluate the association between occupational exposure to benzene and risk of NHL in this population-based cohort consisting of Chinese women living in Shanghai.

## Methods

*Study population and outcome ascertainment*. Details concerning the study population enrolled in the Shanghai Women’s Health Study (SWHS) have been described previously (Zheng et al. 2005). Briefly, the study is a prospective cohort of 74,942 women living in urban Shanghai who were enrolled between the ages of 40 and 70 years, with subject enrollment occurring between December 1996 and May 2000. Eligible women for the study were identified from a roster of all women from the resident offices in the study communities and were approached by a trained interviewer and a local community health worker. The overall participation rate for the study was 92.7% (defined as women who agreed to participate and completed the baseline survey among all eligible women who lived in the study communities during the time period of the baseline survey). Enrolled women completed a lifetime occupational history questionnaire that assessed job title, name of the work place, type of process or business, description of work tasks, and employment dates for all jobs held for at least 1 year. Other components of the questionnaire included an assessment of demographic characteristics, personal lifestyle habits, medical history, and a residential history (Zheng et al. 2005). The Standard Chinese Classification of Industries and Occupations for the third national census (World Bank 2014) was used by investigators from the Shanghai Cancer Institute to code and standardize each of the employer types and free-text job titles obtained from the occupational history questionnaire (Friesen et al. 2012).

All hospitals in Shanghai are legally required to notify the Shanghai Cancer Registry (Shanghai Cancer Institute 2015) of all newly diagnosed cancer cases. Thus, identification of disease outcomes for the study, including incident cancers and vital status, was conducted through annual record linkage to these population-based Shanghai cancer and vital statistics registries. In addition, in-person follow-up study surveys that have taken place every 2 to 3 years have collected disease and hospital information. Medical charts were reviewed from each diagnostic hospital to verify the cancer diagnoses and to collect information on tumor characteristics. All cancer site information was based on the *International Classification of Diseases, Ninth Revision* (ICD-9). All participants provided informed consent, and the study was approved by the institutional review boards of all participating institutions.

*Exposure assessment*. The exposure assessment used a benzene JEM that was composed of two separate JEMs, one based on occupation and one based on industry. The JEM was calibrated with short-term area benzene exposure measurements in Shanghai factories and has been described in detail elsewhere (Friesen et al. 2012). Briefly, the JEM using the same industry and job coding system as the questionnaire data was developed based on experts’ rating of the probability and intensity of benzene exposure using an ordinal scale (coded 0–3) for 1980, the median year of employment in the study population. The probability ratings were assigned based on the estimated proportion of workers exposed to benzene, with scores of 0 to 3 corresponding to no exposure, < 5% exposed, 5–50% exposed, and > 50% exposed, respectively. Intensity ratings were assigned based on the maximum allowable concentration (MAC) for occupational benzene exposure in China in the year 1980 (i.e., 40 mg/m^3^), with scores of 0 to 3 corresponding to very low or negligible exposure, above background but < 10% of the MAC, 10–100% of the MAC, and exposures above the MAC, respectively.

The benzene exposure database consisted of short-term area air measurements (*n* = 70,937) that were collected as part of health and safety inspections from factories in Shanghai that fell under the Shanghai Centers for Disease Control (CDC) inspection program. Measurements were collected over the period 1954–2000 by the Shanghai CDC and included the sampling date, factory name, and type of industry and job, all of which were abstracted from records maintained at municipal and district stations. Each measurement was coded to its relevant industry and occupational classification, using the same classification system as used in the occupational histories and JEM. Information pertaining to the analytical method that was used to measure benzene exposure was not available in the database. As described previously (Friesen et al. 2012), in sensitivity analyses the measurements were calibrated to a single measurement method based on the prevalence of syringe measurement and gas chromatography methods over time and the magnitude of the exposure difference between methods observed in another Shanghai study. The rank order of the benzene estimates derived from separate models adjusting or not adjusting for the analytical method was demonstrated to be very highly correlated. The benzene measurement data were combined with the JEMs to derive cumulative exposure estimates (milligrams per cubic meter) for each woman as further described below.

The job/industry–specific benzene exposure estimates for each three-digit job/industry code were derived using mixed-effects modeling that combined both the JEM and the inspection measurements, as described elsewhere (Friesen et al. 2012). Briefly, the estimated geometric mean exposure levels were derived from the fixed-effect parameters for year (incorporated as a b-spline term) and JEM intensity ratings (incorporated as ordinal metrics, 0–3 scale) and from the best linear unbiased prediction estimates for the random-effects of job group, and industry class nested within job group, which allowed for job- and industry-specific deviations from the pooled estimates associated with the JEM ratings (Friesen et al. 2012). Thus, this framework provided a mechanism to account for a 20-fold difference in exposure concentrations between job/industry groups, as well as a 13-fold difference in exposure concentrations between 1954 and 2000, that would not have been possible to capture using the exposure matrices only (Friesen et al. 2012).

For each woman, we applied the time-varying exposure levels predicted by the models above to each year of a job in her work history that was classified as exposed, with exposed jobs defined as those with a job code probability rating of 3 (i.e., > 50% exposed), or as jobs with an industry code probability rating of 3 (i.e., > 50% exposed) and a job code probability rating ≥ 1 (i.e., > 0% exposed). All other jobs were classified as unexposed and assigned a benzene exposure level of zero (Friesen et al. 2012). Occupational histories were available for each woman up until the year of entry into the study.

*Statistical analysis*. Analyses included all women currently enrolled in the SWHS with no prevalent cancer at baseline and a valid occupational history. A total of 73,087 women were included in the analyses, with follow-up for each woman starting at the time of completion of the baseline questionnaire. To evaluate the association between the job-industry–specific benzene exposure metrics and risk of NHL (ICD-9 codes 200, 202, 204.0–204.1), we estimated hazard ratios (HR) and calculated 95% confidence intervals (CIs) using Cox proportional hazard models using entry and exit age as the underlying time factor. Of the total of 102 patients diagnosed with primary incident NHL during follow-up, 24 were ever exposed to benzene. Results are presented for NHL overall because of the lack of detailed histology data for NHL subtypes (i.e., 73% of the 102 cases are classified as ICD-9 code 202.8, “other lymphoma”). Exit age for the analyses was based on the date of NHL diagnosis, the date of death, or the last follow-up date, whichever event came first. Given the previously established relationship between benzene exposure and risk of myeloid leukemia (IARC 2012), we similarly evaluated the association between myeloid leukemia and the benzene exposure metrics, although the number of cases in our cohort was small (*n* = 33 overall, 4 exposed).

Metrics of occupational benzene exposure evaluated in the study included ever/never exposure, duration of exposure in years, and cumulative exposure in milligrams per cubic meter for total years (mg/m^3^-years). Women were classified as ever exposed if they had any job/industry combination in their occupational history that was classified as exposed according to our criteria, regardless of duration. Total exposure duration was calculated by summing the number of exposed years for each woman (i.e., the number of years spent working in jobs classified as exposed). Cumulative exposure was calculated by summing all yearly exposure estimates for exposed jobs across the full occupational history for each subject. For the duration and cumulative exposure metrics, indicator variables based on the tertiles of exposure in the exposed population were included in the Cox regression models, with women having no exposure used as the reference group in all analyses. For these continuous exposure metrics, the highest tertile was further subdivided based on the intracategory median to evaluate associations with NHL across a greater range of exposure levels. We tested for a linear trend in the HR for each of the benzene exposure metrics where applicable by including a continuous variable in the model representing the median values of each of the exposure tertiles. The assumption of proportional hazards was evaluated and verified by a graphical check on the log cumulative hazard versus time and by modeling a product term between the time-scale for the model (attained age) and the time-fixed benzene exposure variables at baseline.

For each of the metrics described above, results are presented from a model adjusted only for age (i.e., using age as the time-scale) and a model additionally adjusted for other covariates. The fully adjusted models included categorical variables for education, body mass index (BMI), ever smoking, and ever use of alcohol. All models were stratified by birth cohort in 5-year intervals to account for possible calendar effects. For the myeloid leukemia analyses, results from a model adjusted only for age and a model further adjusted for ever smoking and education are presented for ever exposure, duration (based on the median and tertiles) and cumulative exposure based on the median. Further, we evaluated the association between cumulative benzene exposure and NHL risk using a 5- and 10-year lag by treating cumulative exposure as a time-varying explanatory variable in the model and using age as the time scale, as implemented using the stcox procedure in Stata version 13.1 (StataCorp). All other analyses were conducted using SAS version 9.3 (SAS Institute Inc.), with a two-sided α = 0.05 considered statistically significant.

## Results

The demographic characteristics at baseline of the 73,087 subjects included in the analyses both overall and stratified by benzene exposure status, are shown in Table 1. Overall, the mean age of women in the cohort at baseline was 52 years, and the mean attained age at the end of follow-up was 64 years. The mean BMI at baseline among all women was 23 kg/m^2^, and most women did not report ever smoking or alcohol use (≥ 97%). These characteristics were similar in workers exposed to benzene and in the unexposed workers (Table 1). About 86% of women had less than a college education.

**Table 1:** Demographic characteristics of women enrolled in the Shanghai Women’s Health Study included in the benzene exposure analysis.

Characteristic	Total *n *= 73,087	Exposed *n *= 10,788	Not exposed *n *= 62,299
Age at baseline (mean ± SD)	52 ± 9	51 ± 9	52 ± 9
Attained age after follow-up (mean ± SD)	64 ± 9	63 ± 9	64 ± 9
BMI at baseline (mean ± SD)	23 ± 3	23 ± 3	23 ± 3
Year of birth category [*n* (%)]
1927–1930	4,328 (6)	584 (5)	3,744 (6)
1931–1935	10,775 (15)	1,486 (14)	9,289 (15)
1936–1940	7,819 (11)	1,171 (11)	6,648 (11)
1941–1945	8,065 (11)	1,059 (10)	7,006 (11)
1946–1950	12,674 (17)	1,736 (16)	10,938 (18)
1951–1955	18,825 (26)	2,913 (27)	15,912 (26)
1956–1960	10,601 (15)	1,839 (17)	8,762 (14)
Current smoker at baseline [*n* (%)]
Yes	1,713 (2)	298 (3)	1,415 (2)
No	71,373 (98)	10,490 (97)	60,883 (98)
Missing	1 (< 1)	0 (0)	1 (< 1)
Alcohol use at baseline [*n* (%)]
Yes	1,650 (2)	275 (3)	1,375 (2)
No	71,437 (98)	10,513 (97)	60,924 (98)
Education at baseline [*n* (%)]
Elementary or less	15,482 (21)	2,677 (25)	12,805 (21)
Middle school	27,230 (37)	4,851 (45)	22,379 (36)
High school	20,465 (28)	2,608 (24)	17,857 (29)
College or higher	9,897 (14)	649 (6)	9,248 (15)
Missing	13 (< 1)	3 (< 1)	10 (< 1)

Of the 73,087 women included in the analysis, 15% (*n* = 10,788) were ever exposed to benzene. Approximately 85% of ever-exposed women did not have an exposed job during the year of cohort entry, which is consistent with the decreasing distribution of exposed person-years from 1980 to 2000 for women in the cohort. The median cumulative exposure level and duration of exposure in exposed workers was 59 mg/m^3^-years (75th percentile: 136 mg/m^3^-years; 95th percentile: 491 mg/m^3^-years) and 17 years, respectively. Among workers exposed to benzene, the predominant industries of employment in terms of person-years from the occupational histories included manufacturing of rubber products (13%), organic chemicals (10%), motor vehicles (8%), miscellaneous electronics (7%), and television sets and audio equipment (6%). The majority of exposed NHL cases (~ 71%) were exposed before 1990.

The mean and median length of time between first year of exposure to benzene and NHL diagnosis were both approximately 38 years (range, 13–59 years). Associations with NHL for each of the benzene exposure metrics are shown in Table 2 for the models adjusted only for age, as well as for the fully adjusted models. For all of the benzene metrics evaluated, further adjustment of the models for education, ever smoking, ever use of alcohol, and BMI had little or no impact on the results for NHL (Table 2, model 2); therefore, HRs and 95% CIs described here are based on the models with age as the time factor and no further adjustment for covariates. Women ever exposed to benzene had a significantly higher risk of NHL relative to unexposed women (HR = 1.87, 95% CI: 1.19, 2.96).

**Table 2:** Associations between the job/industry–specific benzene exposure metrics and non-Hodgkin lymphoma.

Exposure metric	No. of participants	Cases	Unadjusted model^*a*^		Adjusted model^*b*^	
			HR (95% CI)	*p*-Value	HR (95% CI)	*p*-Value
Ever exposure^*c*^
Unexposed	62,299	78	1.0 (reference)		1.0 (reference)
Exposed	10,788	24	1.87 (1.19, 2.96)	0.007	1.86 (1.17, 2.96)	0.009
Exposure duration
Unexposed	62,299	78	1.0 (reference)		1.0 (reference)
Tertile 1 (1–11 years)	3,698	6	1.44 (0.63, 3.31)	0.39	1.45 (0.63, 3.32)	0.39
Tertile 2 (12–21 years)	3,562	8	2.10 (1.01, 4.35)	0.047	2.09 (1.00, 4.36)	0.049
Tertile 3 (> 21 years)	3,528	10	2.07 (1.07, 4.01)	0.03	2.04 (1.05, 3.97)	0.04
*p*_trend_^*d*^				0.006		0.007
22–27 years	1,846	3	1.38 (0.44, 4.37)	0.59	1.37 (0.43, 4.34)	0.60
> 27 years	1,682	7	2.65 (1.21, 5.77)	0.01	2.60 (1.19, 5.69)	0.02
Cumulative exposure (mg/m^3^-years)
Unexposed	62,299	78	1.0 (reference)		1.0 (reference)
Tertile 1 (≤ 35.2 mg/m^3^-years)	3,560	3	0.93 (0.29, 2.96)	0.90	0.92 (0.29, 2.94)	0.89
Tertile 2 (35.21–102.4 mg/m^3^-years)	3,667	9	2.22 (1.12, 4.44)	0.02	2.20 (1.10, 4.41)	0.03
Tertile 3 (> 102.4 mg/m^3^-years)	3,561	12	2.16 (1.17, 3.98)	0.01	2.16 (1.17, 4.00)	0.01
*p*_trend_^*d*^				0.005		0.006
102.41–196.9 mg/m^3^-years	1,780	5	1.98 (0.80, 4.91)	0.14	1.97 (0.80, 4.89)	0.14
> 196.9 mg/m^3^-years	1,781	7	2.31 (1.06, 5.02)	0.03	2.33 (1.07, 5.08)	0.03
^***a***^HRs (hazard ratios) and 95% CIs estimated from Cox proportional hazard models with entry and exit age as the time scale. ^***b***^HRs and 95% CIs estimated from Cox proportional hazard models with entry and exit age as the time scale, and further adjusted for ever smoking, ever use of alcohol, BMI, and education. ^***c***^Women were defined as ever exposed if they had any job/industry combination in their occupational history that was classified as exposed according to our specified criteria, regardless of duration. ^***d***^A linear trend in the HR for each of the benzene exposure metrics was evaluated by including a continuous variable in the model representing the median values of each of the exposure tertiles.						

Significantly higher associations of similar magnitude were apparent for women in the highest two duration tertiles compared with unexposed women (tertile 2, HR = 2.10, 95% CI: 1.01, 4.35; tertile 3, HR = 2.07, 95% CI: 1.07, 4.01; *p*_trend_ = 0.006), but the estimated risk for women in the lowest tertile was not significantly different from unexposed women (Table 2). Further stratification of the top duration tertile (*n* = 10 exposed cases) based on the intracategory median revealed that the majority of the cases within this tertile were exposed to benzene for > 27 years. A significantly higher risk of NHL was observed in the women who were exposed for the longest period of time (i.e., > 27 years; HR = 2.65, 95% CI: 1.21, 5.77), but no significantly higher risk was observed in women in the lower half of the top tertile, which only included three exposed cases (Table 2).

Compared with unexposed women, the risk of NHL was also significantly higher for women in the two highest tertiles of cumulative benzene exposure (tertile 2, HR = 2.22, 95% CI: 1.12, 4.44; tertile 3, HR = 2.16, 95% CI: 1.17, 3.98; *p*_trend_ = 0.005), whereas there was no significant association for women in the lowest cumulative exposure tertile (Table 2). Similar to results for the duration metric, further stratification of the highest tertile showed that the largest magnitude of risk for NHL was in women with cumulative exposures greater than the median exposure (HR = 2.31, 95% CI: 1.06, 5.02) within the highest tertile, compared to unexposed women (Table 2). A similar though nonsignificant higher risk of NHL was observed for women with exposure levels in the lower half of the top tertile (HR = 1.98, 95% CI: 0.80, 4.91). Further, results for cumulative exposure were generally consistent in analyses using a 5- and 10-year lag, with the highest risks of similar magnitude observed in the second (5-year lag, HR = 2.08, 95% CI: 1.00, 4.33; 10-year lag, HR = 2.16, 95% CI: 1.04, 4.49) and third tertiles (5-year lag, HR = 2.16, 95% CI: 1.17, 4.00; 10-year lag, HR = 2.04, 95% CI: 1.08, 3.86) compared with unexposed women, whereas there was no significant association with NHL among women in the first tertile for either analysis. Significant trends were observed in both analyses (*p*_trends_ = 0.007 and 0.02 for 5- and 10-year lags, respectively) (see Supplemental Material, Table S1).

A higher risk of myeloid leukemia was not observed based on four women ever exposed to benzene (see Supplemental Material, Table S2). However, the four benzene-exposed myeloid leukemia cases in our cohort were all highly exposed (i.e., greater than median cumulative exposure and exposed > 21 years) and a higher risk of myeloid leukemia was observed for women in the highest exposure duration tertile (HR = 2.62, 95% CI: 0.92, 7.50) and for workers with cumulative exposure levels higher than the median of 59 mg/m^3^-years (HR = 1.65, 95% CI: 0.58, 4.71). These risks were slightly attenuated after further adjustment for ever smoking and education (see Supplemental Material, Table S2).

## Discussion

To our knowledge, this is the first study to investigate the risk of NHL in relation to benzene exposure in the context of a population-based prospective cohort study of women with diverse occupational histories and job tasks. We observed that women who were ever exposed to benzene had a significantly higher risk of NHL compared with unexposed women, and significantly increased risks were also observed among women in the highest two tertiles of exposure duration and cumulative exposure. Our study, which combined job- and industry-exposure matrices and short-term area air measurements in Shanghai factories, provides evidence that occupational benzene exposure is associated with NHL among these women from Shanghai.

The lymphomagenic potential of benzene exposure has been the subject of much debate. A recent comprehensive evaluation by IARC concluded that there is evidence in humans for a positive association between benzene and NHL overall, as well as for acute lymphocytic leukemia (ALL), CLL, and multiple myeloma (IARC 2012). Notably, a relative risk of 3 was previously observed for NHL in relation to ever exposure to benzene in a cohort of Chinese workers (Hayes et al. 1997), whereas the majority of other occupational cohorts conducted to date have not observed significantly higher associations for NHL (IARC 2012). The estimated benzene exposure levels in our study were lower, on average, than those reported in the previous Chinese occupational cohort, which observed the highest risk for NHL associated with cumulative benzene exposures > 100 ppm-years, but were higher, on average, compared to several cohorts conducted in North America and Australia, which reported mean or median cumulative benzene exposures generally < 10 ppm-years (Collins et al. 2003; Glass et al. 2003; Schnatter et al. 1996). It is also notable that most previous cohorts have not included lymphoid leukemia cases in their NHL case definition, whereas we included cases of both CLL/small lymphocytic lymphoma and ALL, which are grouped with NHL in the most recent classifications (Turner et al. 2010). Differences between cohorts with respect to evaluating NHL incidence versus mortality also present challenges when comparing findings across studies. Recent case–control studies conducted in Caucasian populations have also provided some suggestive evidence for an association between benzene exposure with either NHL overall or with specific NHL subtypes (Costantini et al. 2008; Dryver et al. 2004; Fabbro-Peray et al. 2001; Miligi et al. 2006; Wang et al. 2009). A previous hospital-based case–control study of 649 NHL cases and 1,298 controls in Shanghai found a significantly higher risk of follicular lymphoma for ever exposure to benzene based on a limited number of cases but not for other subtypes or NHL overall (Wong et al. 2010).

A particular challenge in the evaluation of factors associated with NHL risk is the increasing evidence of etiologic heterogeneity for specific NHL subtypes (Morton et al. 2008). To that end, a recent meta-analysis of occupational cohort studies evaluating benzene exposure and cancer risk found evidence for an association with CLL and ALL, while an association with NHL overall was less pronounced (Vlaanderen et al. 2011). Further, evidence from some case–control studies has indicated that solvent use more generally may be associated with some NHL subtypes but not others (Cocco et al. 2010; Wang et al. 2009). Because we lacked detailed histology data on the NHL cases in our cohort, we were unable to evaluate NHL subtype-specific associations.

Exposure to benzene has been demonstrated in animal studies to result in development of lymphomas, including in one study of Trp53-deficient mice that also observed an increase in benzene-induced AML (IARC 2012; Kawasaki et al. 2009). The IARC working group evaluating benzene exposure concluded that there are two probable mechanisms of benzene-induced lymphomagenesis in humans, namely, immunosuppression leading to decreased immunosurveillance and chromosomal rearrangements (IARC 2012). In support of these conclusions, studies of benzene have shown a variety of genotoxic effects in humans resulting from exposure, including both structural and numerical chromosomal abnormalities; these genotoxic effects appear to affect the lymphocytes of exposed populations (IARC 2012; Zhang et al. 2002). Previous molecular epidemiologic investigations conducted in the Chinese population in particular, including among healthy workers in Shanghai (Rothman et al. 1996), have demonstrated that benzene is associated with cytogenetic abnormalities in cultured peripheral lymphocytes, which are frequently associated with lymphomas, including the long arm deletion of chromosome 6 (Zhang et al. 2007). Further, we have shown among workers in China that benzene exposure is associated with increased mitochondrial DNA (mtDNA) copy number (Shen et al. 2008). Interestingly, increased mtDNA copy number was positively associated with NHL in a dose-dependent manner in a prospective cohort of Finnish men, and thus the effect of benzene on this marker may represent an early biologic effect that is relevant to the subsequent development of NHL (Lan et al. 2008). Finally, a cross-sectional study of occupationally exposed workers in China found a decrease in lymphocyte cell counts including CD4^+^ T cells, which has been associated with an increased risk of NHL, at benzene exposures as low as < 1 ppm, with greater reductions observed for higher levels of benzene (Lan et al. 2004). The inverse association of benzene exposure on levels of CD4^+^ T cells is particularly notable given the established relationship between immune deficiency, various immune disorders, and NHL risk (Grulich et al. 2007). The increased risk of NHL associated with immune deficiency may result from loss of viral control resulting from reduced immunosurveillance or due to dysregulation of B-lymphocyte activity (Grulich et al. 2007). Although the relationship between subclinical immune deficiency and NHL is not as clear, the apparent lack of a threshold between levels of immunodeficiency and subsequent NHL risk (Grulich et al. 2007), as well as more recent prospective studies suggesting that subclinical alterations in various immune markers are associated with NHL (De Roos et al. 2012; Purdue et al. 2013), provides evidence for a role of more subtle immune alterations in lymphomagenesis.

The most recent IARC assessment of benzene also confirmed that there continues to be sufficient evidence in humans for an association with AML (IARC 2012). The number of exposed myeloid leukemia cases in our cohort was small (*n* = 4), and only 2 of these were AML; therefore, we had limited ability to evaluate this association. Because occupational benzene exposure had ceased for the vast majority of cohort members ever exposed to benzene by the time of enrollment into the cohort, it is possible that deaths due to benzene-related AML had already taken place among people living within the cohort catchment area well before enrollment began. This possibility is based on some evidence suggesting that benzene exposure during the 5 years prior to diagnosis may be the most important for determining risk for benzene-related AML (Hayes et al. 1997).

A strength of our population-based study of Chinese women was the use of an exposure assessment framework with improved contrast in exposure levels across time and between jobs/industries compared with using the JEM alone (Friesen et al. 2012). Second, we used stringent definitions for defining exposure, and our *a priori* criteria were chosen to maximize specificity in the exposure assessment, as recommended previously (Kromhout and Vermeulen 2001). A further strength of our study was the acquisition of a full occupational history for women in the cohort and the availability of questionnaire data for women in the SWHS, although we lacked data on potential coexposures in the workplace and therefore cannot exclude the possibility of confounding from other chemical exposures. Nevertheless, the SWHS includes women with diverse job tasks across a variety of industries; thus, the common use of a confounding chemical(s) may be less likely, compared with occupational cohorts that include subjects employed in only one or a few industries with similar exposure patterns.

Given that the exposure measurements from the Shanghai database used in our framework reflected short-term area samples that were collected for health and safety inspection purposes, as opposed to using full-shift personal monitors, some degree of exposure misclassification is likely, as has been discussed in detail elsewhere (Friesen et al. 2012). The use of area measurements could result in either an over- or underestimation of an individual worker’s exposure level. The exposure measurements used in our study were from factories included in the Shanghai CDC inspection program, which did not include all factories in Shanghai. However, there were a relatively large number of measurements from this database that were below the limit of detection (~ 50%). Moreover, our exposure estimates were, on average, lower than those from some previous studies conducted in China that measured benzene exposure in factories under investigation for benzene-poisoning events or factories with suspected high-level exposures under consideration for use in molecular epidemiology studies (Friesen et al. 2012). These observations provide some evidence that the database reflected a wide range of exposure scenarios rather than worst-case scenarios only. Comparisons between the experts’ intensity estimates and our model predictions for the year 1980 found that the predicted levels for job intensity ratings of 1, 2, and 3 were 4 mg/m^3^, 6 mg/m^3^, and 11 mg/m^3^, respectively, which were consistent with the expert cut points for intensity ratings of 1 [arithmetic mean (AM), < 4 mg/m^3^] and 2 (AM, 4–40 mg/m^3^) but were lower than the cut point for intensity rating 3 (AM > 40 mg/m^3^) (Friesen et al. 2012). This may not be surprising because the measurements would reflect to some extent both the intensity of the exposure and the probability of exposure, whereas the cut points were based on intensity of exposure only. However, our exposure assessment approach that combined the JEM with the benzene exposure measurements provided a mechanism to calibrate the ratings to a concentration scale and across time, and thus the measurement-driven values—rather than the theoretical cut points—were used.

Moreover, the measurements applied in our study were not directly associated with any individual subject but rather were used in the models to estimate geometric mean exposure levels for a given job/industry. Berkson error resulting from the assignment of group-level estimates to the individual level has been demonstrated to result in minimal bias to the risk estimates in the presence of small measurement error (Küchenhoff et al. 2007). Second, because occupational history data in the SWHS was not available after the cohort entry year, the estimated benzene exposure levels would therefore slightly underestimate cumulative exposure and duration of exposure for the minority (*n* = 1,822) of benzene-exposed women who still had an exposed job at the start of follow-up, if these women had continued exposure during the follow-up period. For these reasons, we placed greater weight on reporting associations with NHL based on the rank-order exposure tertiles rather than emphasizing specific exposure levels in mg/m^3^-years. This analytic approach would be less sensitive to slight underestimates in exposure relative to associations based on continuous analyses. Moreover, this approach combined with the decreasing person-years and concentration of benzene exposure over time observed in our study (Friesen et al. 2012), as well as the fact that elevated associations with NHL were still apparent in the lagged analyses, suggest that any continued exposure during the follow-up period is unlikely to have materially changed our conclusions. Third, the limited histology data in the cohort precluded an evaluation of the risk of specific NHL subtypes in relation to benzene exposure. Thus, given that subtype-specific associations have been suggested in some previous case–control studies (Cocco et al. 2010; Wang et al. 2009), future studies that are able to obtain tumor samples on a high percentage of cases and that are able to conduct pathology review are needed to determine whether associations with specific NHL subtypes may be driving the observed association with overall NHL risk. Finally, given that NHL is a rare tumor in China, another limitation is the relatively small number of NHL cases in the cohort, which contributed to relatively broad confidence intervals associated with the observed effect estimates. Nonetheless, we note that the number of benzene-exposed cases is similar or larger to those of other cohorts that have evaluated this association (IARC 2012); despite the relatively small number of exposed cases, we observed significant elevations in NHL risk for three different benzene metrics, including ever exposure.

## Conclusions

We estimated the relative risk of NHL in a population-based cohort study of Chinese women living in Shanghai using an exposure assessment framework that maximized information from two sources of exposure information. We found evidence for an increased risk of NHL for women ever exposed to benzene and for those with higher cumulative exposure levels and durations of occupational benzene exposure. Our findings are consistent with several previous studies that have found an elevated risk of NHL overall or of specific NHL subtypes associated with benzene exposure, and they provide additional evidence that occupational exposure to benzene is associated with NHL risk.

## Supplemental Material

(213 KB) PDF
