# Supplementary material for: Occupational Exposure to Benzene and Non-Hodgkin Lymphoma in a Population-Based Cohort: The Shanghai Women’s Health Study
Source: Environ Health Perspect. 2015 Mar 6;123(10):971–7. doi: 10.1289/ehp.1408307 (PMC4590744; doi:10.1289/ehp.1408307)
Supplement: (213 KB) PDF [file ehp.1408307.s001.acco.pdf]

Environ Health Perspect
doi: 10.1289/ehp.1408307

Note to Readers: ***EHP*** **strives to ensure that all journal content is accessible to all readers. However, some figures and Supplemental Material published in** ***EHP*** **articles may not conform to 508 standards due to the complexity of the information being presented. If you need assistance accessing journal content, please contact ehp508@niehs.nih.gov. Our staff will work with you to assess and meet your accessibility needs within 3 working days.**

Supplemental Material

Occupational Exposure to Benzene and Non-Hodgkin Lymphoma in a Population-Based Cohort: The Shanghai Women's Health Study

Bryan A. Bassig, Melissa C. Friesen, Roel Vermeulen, Xiao-Ou Shu, Mark P. Purdue, Patricia A. Stewart, Yong-Bing Xiang, Wong-Ho Chow, Tongzhang Zheng, Bu-Tian Ji, Gong Yang, Martha S. Linet, Wei Hu, Heping Zhang, Wei Zheng, Yu-Tang Gao, Nathaniel Rothman, and Qing Lan

Table of Contents

Table S1. Associations between cumulative benzene exposure and NHL with 5- and 10-year lag of exposure.

Table S2. Associations between the job/industry specific benzene exposure metrics and myeloid leukemia.

Table S1. Associations between cumulative benzene exposure and NHL with 5- and 10-year lag of exposure.

Exposure Metric	Cases (N)	Person-Years of Follow-up	Hazard Ratio (95% C.I.)[a]	p-value
5-year lag of cumulative exposure (mg/m^3-years)				
Unexposed	78	687,606	1.0 (ref)	
Tertile 1	3	39,217	1.32 (0.48, 3.64)	0.59
Tertile 2	9	39,283	2.08 (1.00, 4.33)	0.05
Tertile 3	12	39,123	2.16 (1.17, 4.00)	0.01
**p trend**				0.007
10-year lag of cumulative exposure (mg/m^3-years)				
Unexposed	78	690,525	1.0 (ref)	
Tertile 1	3	38,406	1.47 (0.53, 4.05)	0.46
Tertile 2	9	38,257	2.16 (1.04, 4.49)	0.039
Tertile 3	12	38,040	2.04 (1.08, 3.86)	0.029
**p trend**				0.02

[a]Lagged cumulative exposure calculated by treating benzene exposure as a time-varying explanatory variable with age as the time scale as implemented using the stcox procedure in Stata v 13.1. Hazard ratios and 95% confidence intervals estimated from Cox proportional hazard models and adjusted for ever smoking, ever use of alcohol, BMI, education, and birth cohort.

Table S2. Associations between the job/industry specific benzene exposure metrics and myeloid leukemia.

**Exposure Metric**	**# Subjects**	**Cases**[a]	**Model 1 (Unadjusted)**[b] **Hazard Ratio (95% CI)**	**p-value**	**Model 2 (Adjusted)**[c] **Hazard Ratio (95% C.I.)**	**p-value**
Ever exposure						
Unexposed	62,299	29	1.0 (ref)		1.0 (ref)	
Exposed	10,788	4	0.83 (0.29, 2.36)	0.72	0.74 (0.26, 2.11)	0.57
Exposure duration						
Unexposed	62,299	29	1.0 (ref)		1.0 (ref)	
≤ median (≤ 17 years)	5,677	0	--	--	--	--
> median (> 17 years)	5,111	4	1.79 (0.63, 5.08)	0.28	1.52 (0.53, 4.38)	0.43
Unexposed	62,299	29	1.0 (ref)		1.0 (ref)	
Tertile 1: (1-11 years)	3,698	0	--	--	--	--
Tertile 2: (12-21 years)	3,562	0	--	--	--	--
Tertile 3: (> 21 years)	3,528	4	2.62 (0.92, 7.50)	0.07	2.23 (0.77, 6.44)	0.14
Cumulative exposure (mg/m^3-years)						
Unexposed	62,299	29	1.0 (ref)		1.0 (ref)	
≤ median (≤ 59.3 mg/m^3-years)	5,394	0	--	--	--	--
> median (> 59.3 mg/m^3-years)	5,394	4	1.65 (0.58, 4.71)	0.35	1.44 (0.50, 4.14)	0.50

[a]33 cases of myeloid leukemia included 17 AML (n=2 exposed), 7 chronic myeloid leukemia (n=1 exposed), 1 acute monocytic leukemia, 2 acute erythroid leukemias, 2 subacute myeloid leukemias, and 4 unspecified myeloid leukemias (n=1 exposed). [b] Hazard ratios and 95% confidence intervals estimated from Cox proportional hazard models with entry and exit age as the time scale. [c]Model 2 further adjusted for ever smoking and education.
